# Maternal Transmission of the *PAX7* Single Nucleotide Polymorphisms among Indian Cleft Trios

**DOI:** 10.1055/s-0042-1760383

**Published:** 2023-01-24

**Authors:** Mahamad Irfanulla Khan, Prashanth C.S, Mohammed S. Mustak, Sheikh Nizamuddin

**Affiliations:** 1 Department of Orthodontics and Dentofacial Orthopedics, The Oxford Dental College, Bangalore, Karnataka, India; 2 Department of Orthodontics and Dentofacial Orthopedics, DAPM R.V Dental College, Bangalore, Karnataka, India; 3 Department of Applied Zoology Mangalore University, Mangalore, Karnataka, India; 4 Department of Urology, Medical Center-University of Freiburg, Germany; 5 German Cancer Consortium (DKTK), German Cancer Research Center (DKFZ), Heidelberg, Germany

**Keywords:** Cleft lip and/or cleft palate, SNP, genotyping, MassARRAY

## Abstract

Cleft lip and/or cleft palate (CL/P) is one of the most common congenital anomalies of the human face with a complex etiology involving multiple genetic and environmental factors. Several studies have shown the association of the paired box 7 ( *PAX7* ) gene with CL/P in different populations worldwide. However, the current literature reveals no reported case-parent trio studies to evaluate the association between the *PAX7* gene and the risk of nonsyndromic cleft lip and/or palate (NSCL/P) in the Indian population. Hence, the purpose of this study was to assess the *PAX7* gene single nucleotide polymorphisms (SNPs) in the etiology of NSCL/P among the Indian cleft trios. Forty Indian case-parent trios of NSCL/P were included. The cases and their parents' genomic DNA were extracted. The SNPs rs9439714, rs1339062, rs6695765, rs742071, and rs618941of the *PAX7* gene were genotyped using the Agena Bio MassARRAY analysis. The allelic transmission disequilibrium test was performed using PLINK software while pair-wise linkage disequilibrium by the Haploview program. The SNP rs9439714 showed evidence of association ( *p* -value = 0.02, odds ratio = 3) with NSCL/P. Considering the parent-of-origin effects, the SNPs rs9439714 and rs618941 showed an excess maternal transmission of allele C at rs9439714 ( *p* -value = 0.05) and G allele at rs618941 ( *p* -value = 0.04). The results of the present study suggested that the SNPs rs9439714 and rs618941 showed an excess maternal transmission of alleles suggestive of the possible role of the *PAX7* gene involvement in the etiology of NSCL/P in the Indian population.

## Introduction

Cleft lip and/or cleft palate (CL/P) is one of the most common congenital birth defects in the human face, with a prevalence of 1 in 700 live births worldwide. 1 The etiology is heterogeneous, with multiple genetic and environmental factors involved in the development of CL/P. 2 3 4 The infants born with CL/P may have complications such as difficulty in feeding, esthetics, and other psychological problems. 5 The World Health Organization has recognized and included CL/P in their Global Burden of Disease initiative. 6

Clefts are classified as nonsyndromic or syndromic based on whether the child has other additional physical or cognitive deformities. 7 The incidence of CL/P varies according to race, geography, ethnicity, and socioeconomic status. 8 9 The highest prevalence rate is found in Asians and American Indians (1:500), the intermediate in Europeans (1:1,000), and the lowest in Africans (1:2,500). 10 In India, the incidence of clefts ranges between 1:800 and 1:1,000, with three infants born with some form of cleft every hour. 11 Consanguinity is a risk factor for nonsyndromic CL/P in the Indian population, according to a 13-year retrospective study from a cleft center. 12

The paired box 7 ( *PAX7* ) gene is a member of the paired box ( *PAX* ) family, located at 1p36.13, and encodes specific DNA-binding transcription factors. *PAX7* is involved in neural crest development, myogenesis, and maxilla development in humans. A literature search showed deformity of the maxilla in the mutant mice, thus confirming *PAX7* role in craniofacial defects such as cleft palate. 13

Case-parent trio studies are a popular alternative to population-based case-control studies in genetics. They are useful for studying the transmission of genetic variants between parents and children and how genetic variants differ between affected and unaffected individuals within a family. 14 The advantages of case-parent trio studies include robustness in sample collection, studying the parent-of-origin (PoO) effects and correct mutations. 15 It is critical to consider the PoO effects 16 while studying craniofacial deformities such as CL/P, where maternal genotype controls the in-utero environment of the developing fetus and separates maternal genotypic effects from imprinting effects. 17 Furthermore, studies that include maternal and fetal genotypes and gestational environmental exposures will reveal more about the gene–environment interaction. 18

A population-based cohort study supported that maternal genotype contributed to the development of CL/P in the offspring. 19 If the maternal genotypes play a major factor in the development of congenital malformations during pregnancy, one expects the mother-offspring recurrence rate to be higher than the father-offspring recurrence rate. 20

The case-parent trio design studies are very rare in India. The current literature reveals no reported case-parent trio studies to evaluate the association between the *PAX7* gene and the risk of nonsyndromic cleft lip and/or palate (NSCL/P) in the Indian population. Hence, we selected these high-risk single nucleotide polymorphisms (SNPs) from the literature (previous genome-wide association studies [GWAS]) to know whether these *PAX7* gene SNPs are involved in the etiology of NSCL/P considering the PoO effects.

## Materials and Methods

### Study Population and Ethical approval

The Institutional Review Board of the DAPM RV Dental College, Bangalore (IRB No. 230/Vol-2/2017) approved the research, and it was performed as per Helsinki's declaration for experiments involving human subjects.

The study included 40 case-parent trios (120 subjects) of NSCL/P. A geneticist clinically assessed the case-parent trios to rule out syndromic cases. Patients with other congenital malformations and syndromes were excluded. All participants were informed about the study, and written informed consent was obtained from all patients and parents. The phenotypic features and the demographic distribution of the samples are presented in Table 1 . The study population's mean age and standard deviation included cleft patients: 6.62 ± 5.30 years, fathers: 36.07  ± 6.42 years, and mothers: 30.37  ± 5.45 years.

**Table 1:** Demographic distribution of the samples

Cleft type	Male	Female	Total
NSCLO	6	7	13
NSCLP	10	11	21
NSCPO		6	6
			40

**Abbreviations:** NSCLO, nonsyndromic cleft lip only; NSCLP, nonsyndromic cleft lip with cleft palate; NSCPO, non-syndromic cleft palate only.

### Selection of Single Nucleotide Polymorphisms

High-risk *PAX7* SNPs rs9439714, rs1339062, rs6695765, rs742071, and rs618941 ( Table 2 ) were obtained from the literature and the National Centre for Biotechnology Information dbSNP database ( http://www.ncbi.nlm.nih.gov/SNP/ ).

**Table 2:** SNP information of the *PAX7* gene tested

Gene	SNP	Genomic position	Consequence type	Alleles	Ancestral allele	MAF
*PAX7*	rs9439714	1:18649995	Intron variant	T/C	T	0.47
*PAX7*	rs1339062	1:18651988	Intron variant	T/C	C	0.45
*PAX7*	rs6695765	1:18652826	Intron variant	T/A/C	C	0.49
*PAX7*	rs742071	1:18653380	Intron variant	G/T	G	0.48
*PAX7*	rs618941	1:18679658	Intron variant	G/A/C	A	0.39

**Abbreviations:** A, adenine; C, cytosine; G, guanine; MAF, minor allele frequency; *PAX7* , paired box 7; SNP, single nucleotide polymorphism; T, thymine.

### DNA isolation and Single Nucleotide Polymorphism Genotyping

Three milliliters of peripheral venous blood were collected in the ethylene diamine tetra acetic acid-coated tubes from each affected cleft patient and their parents. Each sample's genomic DNA was isolated from blood lymphocytes using a Qiagen DNA Mini kit (Qiagen GmbH, Hilden, Germany). The selected SNPs were genotyped using the Agena Bio MassARRAY (Agena Bioscience, Inc., San Diego, CA, United States) platform using iPLEX Gold technology, a nonﬂuorescent, highly accurate detection method by matrix-assisted laser desorption/ionization-time of flight mass spectrometry. The genotyping reports were generated using Agena's Spectro Typer 4.0 software (San Diego, United States), and the data obtained were sent for statistical analysis.

### Statistical Analyses

PLINK software (version; 1.09) 21 was used for all statistical analyses. Hardy–Weinberg equilibrium (HWE) was determined for each SNP. We used the allelic transmission disequilibrium test (TDT) 22 to analyze the excess transmission of the target alleles in family-based association analysis, while the PoO effects were analyzed in all case-parent trios using the same PLINK software. The odds ratio (OR) and 95% conﬁdence intervals (CIs) were calculated, and a statistical significance was defined at *p* -value <0.05. Bonferroni corrections were performed for multiple comparisons.

Haplotype identification and haplotype-TDT were performed using the Haploview tool. In addition, Haploview software ( http://www.broad.mit.edu/mpg/haploview/index.php/ ) was used to calculate the pair-wise linkage disequilibrium (LD) for all the SNPs analyzed.

## Results

Table 2 presents the genetic information of each SNP, major/ minor allele, and minor allele frequency. The HWE was determined for the SNPs rs9439714, rs1339062, rs6695765, rs742071, and rs618941 of *PAX7* in all case-parent trios. The allelic TDT analysis ( Table 3 ) showed evidence of the SNP rs9439714 ( *p* -value = 0.02, OR = 3) association with NSCL/P.

**Table 3:** Allelic TDT results of the SNPs

Sl.no	SNP	Genomic position	A1	A2	T	U	OR (95% CI)	CHISQ	*p* -Value
1	**rs9439714**	1:18649995	C	T	15	5	3	5	**0.02**
2	rs1339062	1:18651988	C	T	15	12	1.25	0.33	0.56
3	rs6695765	1:18652826	C	T	15	10	1.5	1	0.31
4	rs742071	1:18653380	T	G	15	8	1.875	2.13	0.14
5	rs618941	1:18679658	G	A	3	8	0.375	2.27	0.13

Abbreviations: A, adenine; A1, minor allele (mutant); A2, major allele (wild allele); C, cytosine; CHISQ, chi square; CI, confidence interval; G, guanine, thymine; OR, odds ratio; SNP, single nucleotide polymorphism; T, minor allele transmitted; U, minor allele untransmitted.

Note: *p* -Value < 0.05 is significant.

Bold SNPs indicates significant association (positive association) with NSCL/P disease whereas, the bold font of *p* -values indicates significant association with *p* -value <0.05 is significant. It helps to understand instantly which SNPs are associated with NSCL/P.

PoO effects were assessed for any significant parental transmission using the TDT ( Table 4 ). The results showed an excess maternal transmission of allele C at rs9439714 ( *p* -value = 0.05) and G allele at rs618941 ( *p* -value = 0.04).

**Table 4:** Parent-of-origin effect of the *PAX7* SNPs

CHR	SNP	A1:A2	Paternal			Maternal			Z score for difference in paternal versus maternal odds ratios	Asymptotic *p* -value for parent-of-origin test
			T: U	Chi-square value	*p* -Value	T: U	Chi-square value	*p* -Value		
1	**rs9439714**	C: T	05:02	1.28	0.25	10:03	3.76	**0.05**	−0.27	0.78
1	rs1339062	C: T	08:05	0.69	0.40	07:07	0	1	0.60	0.54
1	rs6695765	C: T	07:05	0.33	0.56	08:05	0.69	0.40	−0.16	0.87
1	rs742071	T: G	6.5:3.5	0.9	0.34	8.5:4.5	1.23	0.26	−0.01	0.98
1	**rs618941**	G: A	03:04	0.14	0.70	00:04	4	**0.04**	NA	NA

Abbreviations: A, adenine; A1, minor allele (mutant); A2, major allele (wild allele); C, cytosine; CHISQ, chi square; CHR, chromosome number; CI, confidence interval; G, guanine; OR, odds ratio; SNP, single nucleotide polymorphism; T, minor allele transmitted; T, thymine; U, minor allele untransmitted.

Note: *p* -Value < 0.05 is significant.

Bold SNPs indicates significant association (positive association) with NSCL/P disease whereas, the bold font of *p* -values indicates significant association with *p* -value <0.05 is significant. It helps to understand instantly which SNPs are associated with NSCL/P.

**Fig. 1:**
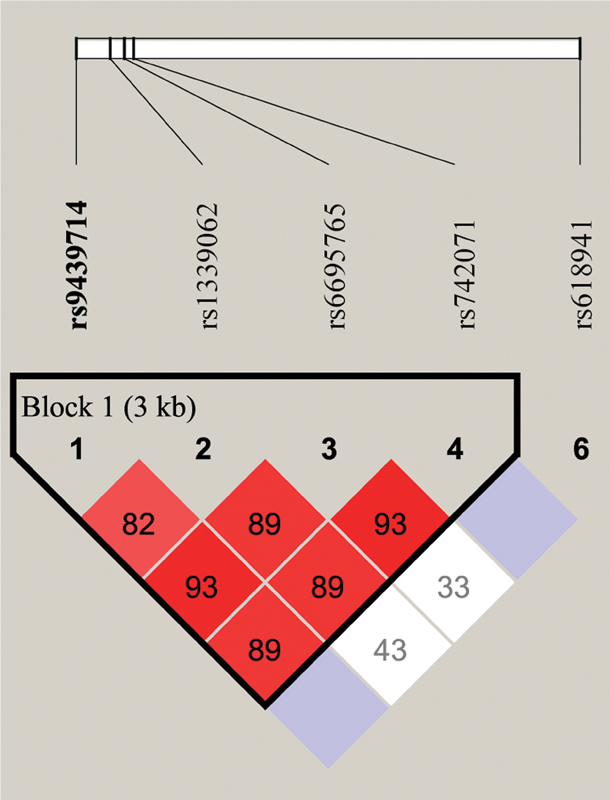
Pair-wise linkage disequilibrium of the *PAX7* SNPs. *PAX7* , paired box 7 gene; SNP, single nucleotide polymorphisms.

The pair-wise LD of the *PAX7* SNPs and the haplotype analysis was assessed based on the TDT results ( Fig. 1 ) using Haploview software. The five SNPs tested are indicated at the top of Fig. 1 , and the numbers in the diamond indicate the percentage of LD between a given pair of SNPs. The haplotype-based frequency and associations of the case-parent trios are shown in Table 5 .

**Table 5:** Haplotype associations of the case-parent trios

Block	Haplotype	Frequency	T: U	Chi square	*p* -Value
Block 1	TTTG	0.554	10.0: 11.0	0.04	0.82
	CCCT	0.203	11.9: 4.0	3.93	0.04
	TTCG	0.093	2.9: 5.0	0.55	0.45
	TCCG	0.046	2.1: 4.0	0.59	0.43
	TCCT	0.037	1.0: 2.0	0.33	0.56
	TCTG	0.020	0.0: 2.0	1.99	0.15
	CTCT	0.019	2.1: 0.0	2.09	0.14
	CTTG	0.019	1.0: 1.0	0.0	1.0

Abbreviations: A, adenine; C, cytosine; G, guanine, thymine; T, minor allele transmitted; U, minor allele untransmitted.

Note: *p* -Value < 0.05 is significant.

## Discussion

The development of NSCL/P is complex and heterogeneous, involving various genetic and environmental risk factors. Despite the complex etiology and pathogenesis of NSCL/P, several genetic factors have been identified by GWAS, linkage studies, and whole-exome sequencing, providing a much more efficient way to detect susceptibility genes and loci causing NSCL/P.

*PAX* genes are involved in craniofacial morphogenesis through cellular proliferation, migration, and the regulation of differentiation programs during embryonic development. *PAX7* gene belong to the *PAX* gene family and plays a critical role during fetal and neural crest development. Several animal studies also demonstrated the deficiency of the *PAX7* gene resulting in the defect in the formation of the nasal cavity, lacrimal bones, and maxilla. The *PAX7* gene, along with *PAX3* , helps to maintain the proliferative cells during the development of fetal muscles of the trunk and limbs. So, any embryological disturbance in the neural crest development may lead to the development of the oral clefts such as cleft lip and palate. 23

Several genomic studies have successfully replicated the associations between *PAX7* and NSCL/P in Singaporean, Korean, Taiwanese, Philippines, Japanese, and Chinese populations. 24 25 26 A family study by Neela et al reported that several genes on the locus 13q33.1–34 were not associated with NSCL/P in the Indian population. 27 Case-parent trio studies are rare in India, and there are no reported case-parent trio studies to evaluate the possible association between the *PAX7* gene and the risk of NSCL/P.

Hence, we employed a case-parent trio study robust against population substructure and more credible than the traditional case-control design while studying congenital anomalies like CL/P. The advantage of trio studies is that they can test maternal versus paternal effects, PoO effects, and correct mutations. 28 When studying congenital defects, it is critical to investigate PoO effects because maternal genotype influences the developing fetuses in-utero environment. The trio design allowed us to compare genotypes and allelic distributions and evaluate the PoO effects. 29 In addition, several studies demonstrated that the maternal genotypes or PoO effects influence the risk of CL/P through interactions with environmental factors or a more complex network of interacting genes. 30

A literature review revealed inconsistencies in the association of several genetic markers in different populations. A genetic marker that has been identified as a risk in one population may not be associated with other populations. The variations in the results could be attributed to geographical differences, epigenetic factors, gene–environment interaction, etc. 31

In the present study, 40 case-parent trios were included. The SNPs rs9439714, rs1339062, rs6695765, rs742071, and rs618941 of *PAX7* were genotyped using the Agena Bio MassARRAY platform.

A case-parent trio study by Sull et al in four different populations (Singaporean, Taiwanese, Korean, and Maryland) showed an excess maternal transmission and a significant association of the SNP rs618941 with NSCL/P. The SNPs of *PAX7* showed a higher maternal OR (transmission) with a greater influence on the risk of development of NSCL/P through maternal effects. 24

A multiethnic genome-wide meta-analysis of the rs9439714 showed a significant association with nonsyndromic orofacial clefts. 32 33 The rs1339062 was strongly associated with NSCL/P ( *p* -value = 2.47E − 05, OR = 1.4) in the Polish population, 34 whereas in an Asian and European trio, the SNP rs1339062 showed no significance. 35

Guo et al found no significance for rs6695765 and rs742071 in a case-control study on nonsyndromic orofacial clefts from northern China. 26 35 The rs742071 is located in the intron of *PAX7* and has been tested extensively in different populations for its possible association with the etiology of NSCL/P. Meta-analysis of GWAS and trio study by Duan et al reported a significant association of rs742071 with nonsyndromic cleft palate only patients among the Western Han Chinese population. 36 37 However, it showed no significant association in our population.

The present study tested the SNPs rs9439714, rs1339062, rs6695765, rs742071, and rs618941 in the etiology of NSCL/P, considering the PoO effects. The results revealed that the SNPs rs9439714 and rs61894 of *PAX7* genes associated with NSCL/P in our population may provide new insight into the previous GWA studies. However, the limitations of our study are relatively smaller sample size and the analysis of only five high-risk SNPs of the *PAX7* .

## Conclusion

The present research showed that the SNP rs9439714 of the *PAX7* is associated with NSCL/P patients. Considering the PoO effects, the SNPs rs9439714 and rs618941 showed an excess maternal transmission of alleles suggestive of the possible role of the *PAX7* gene involvement in the etiology of NSCL/P in the Indian population.

In the light of the above, we recommend further investigations of those tested and other SNPs of the *PAX7* among the Indian population with a larger sample to determine the specific role of these SNPs in the etiology of NSCL/P. In addition, further research using next-generation sequencing is also warranted.
